# Assessing the Environmental Benefits of Extending the Service Lifetime of Solar Photovoltaic Modules

**DOI:** 10.1002/gch2.202300245

**Published:** 2024-07-16

**Authors:** Ahmed Burak Paç, Abdulkerim Gok

**Affiliations:** ^1^ Department of Industrial Engineering Gebze Technical University Kocaeli 41400 Turkey; ^2^ Department of Materials Science and Engineering Gebze Technical University Kocaeli 41400 Turkey

**Keywords:** lamination, life cycle assessment, Monte Carlo simulation, PV module, service lifetime

## Abstract

Requiring no fuel for generation and negligible material/energy for operation and maintenance, photovoltaic (PV) systems have environmental impacts mostly due to the production of modules and the commissioning of power plants. Thus, extending the service lifetime of these systems from 30 to 40 years through an enhanced lamination process for module production potentially reduces environmental impacts per unit energy generated. Life cycle assessment is employed to evaluate the environmental impacts under scenarios for resource utilizations for the new lamination process, operation and maintenance requirements in the extended service lifetime, and degradation rates of the devised modules. Extending the service lifetime significantly reduces environmental impacts across categories, with a 21–27% reduction in global warming potential on the pessimistic and optimistic ends. At least 20% impact reduction is achieved in most impact categories, even under a pessimistic scenario. Considering uncertainty models in the life cycle inventories, samples are generated for scenarios via Monte Carlo simulation, and with significant improvements with large effects in most environmental impact categories, deterministic impact comparisons are supported by ANOVA and Tukey tests. Production strategies for more durable and reliable PV modules have a significant potential in contributing to global sustainability efforts.

## Graphical abstract

PV energy has a crucial role in reshaping the current energy production system to effectively achieve gradual decarbonization and fulfill the goals outlined in the Paris Agreement. Implementing production techniques that may result in PV modules with enhanced service lifetime not only contributes to better performance and lower cost of electricity but also reduces life cycle environmental impacts, and thus, holds substantial promise for supporting worldwide sustainability initiatives. While addressing the reliability issues of PV modules during real‐world operation, comprehensive LCA studies, coupled with sensitivity analysis, can help uncover the most sustainable solutions that can achieve a balance between performance and resource efficiency.

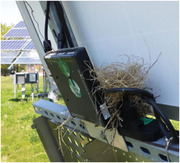

## 1 Introduction

Photovoltaic (PV) modules play a pivotal role in the global shift toward renewable energy. The worldwide deployment of PV systems has consistently risen over the past decade, reached the 1.6 terawatts (TW) level at the end of 2023, which marks a 33% jump from 2022.^[^ ^1^ ^]^ This surge in adoption is attributed to several key factors, including the declining cost of generating electricity from PV systems and their increasing cost‐effectiveness compared to traditional fossil fuels,^[^ ^2^ ^]^ the heightened global consciousness about the benefits of renewable energy for the environment, and the implementation of supportive government incentives and policies aimed at bolstering energy independence.^[^ ^3^ ^]^ Despite this positive trajectory, the growth of PV system installations faces challenges related to the long‐term durability and reliability of PV modules. These issues, which can impact the modules' performance in actual operational conditions and their overall service lifetime, must be addressed to maintain the sustainable expansion of PV installations.^[^ ^4^ ^]^ Contemporary PV modules come with a 30‐year service lifetime performance warranty. Reduced performance as a result of degradation and failure means reduced service lifetime, and thus, higher environmental burden when evaluating life cycle impacts per unit of electricity generated. Extending the service lifetime of PV modules stands at the forefront of sustainable energy solutions, offering a direct pathway to minimizing the environmental impacts of PV energy. Each additional year of reliable module operation not only enhances energy yield but also significantly reduces the cumulative environmental impacts associated with manufacturing, installation, operation, and disposal phases.^[^ ^5^ ^]^ Given that the manufacturing and disposal of PV modules entail considerable energy consumption and material use, extending modules service lifetime directly correlates with a reduction in the frequency of these energy‐intensive processes. This will also allow for a reduction in PV module waste and avoidance of new PV module production, assuming widespread adoption of module designs with extended service lifetimes across the industry. Consequently, by improving the service lifetime of PV modules through advancements in module manufacturing, a marked decrease in life cycle environmental impacts and resource depletion can be achieved. This pivotal relationship underlines the urgency of exploring new strategies that can enhance durability and reliability of modules beyond current standards, thereby delivering more sustainable and environmentally friendly energy solutions.

During real‐world service, PV modules are subjected to a diverse array of environmental stressors, including heat, humidity, ultraviolet (UV) radiation, and mechanical stresses. These conditions lead to a variety of degradation and failure phenomena in PV modules as reviewed in^[^ ^6^ ^]^ and assessed on field deployed modules in.^[^ ^7^ ^]^ Central to the challenge of ensuring the long‐term performance of PV modules is combating the multifaceted nature of module degradation and failure. These issues not only lower performance and curtail the effective service lifetime of PV modules but also amplify their environmental impact by necessitating more frequent replacements. Each component of a PV module plays a crucial role in ensuring its durability and reliability. These components must not only be mutually compatible but also resilient enough to endure the rigors of exposure to climate conditions during service.^[^ ^8^ ^]^ Encapsulant materials for module lamination stands out as one of the most important components in PV modules, offering structural support for mechanical stability, facilitating optical coupling, ensuring electrical isolation to provide electrical safety and prevent leakage currents, and shielding them from environmental conditions to provide physical protection. Ethylene‐Vinyl Acetate (EVA) is the predominant encapsulant material employed in the PV industry to date due to its low cost, high light transmittance, strong adhesion to front glass, adequate mechanical properties, low weight, ease of processing during lamination.^[^ ^9^ ^]^ Nonetheless, a notable challenge arises when moisture penetrates into the module construction, hydrolytically degrading EVA.^[^ ^10^ ^]^ This degradation process generates acetic acid that corrodes cell metallization, thereby increasing series resistance and reducing module performance.^[^ ^11^ ^]^ Degradation of EVA is also associated with changes in the adhesion properties of the encapsulant, deteriorating the bonding between various interfaces, potentially leading to delamination problem.^[^ ^12^ ^]^ Moreover, the polar nature of EVA, along with acetic acid formation upon degradation, increases the module's vulnerability to potential‐induced degradation (PID) by enhancing the transport of ions.^[^ ^13^ ^]^ Furthermore, the performance of PV modules can be compromised by the discoloration of EVA. Marked yellowing or even browning observed in deployed modules signals a severe degradation of EVA.^[^ ^14^ ^]^ This discoloration impedes the transmission of light to the cells, thus lowering the performance. These mechanisms collectively contribute to a decline in module performance, necessitating an exploration of alternative materials and technologies to mitigate their impact and extend service lifetime. The rate at which PV modules degrade during real‐world service also varies, mostly influenced by the quality of module construction and climate conditions. A decline in degradation rates in newer installations post‐2000 is attributed to advancements in manufacturing and installation.^[^ ^15^ ^]^

In recent years, the exploration of new encapsulant materials for module lamination has become a focal point of research, driven by the shortcomings associated with EVA. The primary motivation behind this shift is to improve the durability, reliability, and service lifetime of PV modules. Additionally, there's a concerted effort to lower the cost of energy production and, crucially, to minimize environmental impacts.^[^ ^16^ ^]^ Alternative materials such as thermoplastic polyolefin (TPO), polyolefin elastomer (POE), and ionomers are at the forefront of this research.^[^ ^17^ ^]^ These alternatives boast a polyethylene (PE) backbone but differ in their side groups, featuring elements like acrylates, acrylic acids, or n‐alkanes instead of vinyl acetate.^[^ ^18^ ^]^ This distinction is crucial as it eliminates the formation of acetic acid upon degradation, a notable advantage over EVA. These encapsulants are broadly classified into two groups: non‐crosslinking thermoplastics like TPO, and crosslinking elastomeric materials like POE. Elastomers, including EVA and POE, undergo chemical crosslinking, forming covalent bonds between polymer chains. This crosslinking renders them non‐recyclable. On the other hand, thermoplastics, as TPO and ionomers, bond through physical hydrogen bonds at potentially lower temperatures, facilitating recycling.^[^ ^19^ ^]^ In accelerated weathering experiments conducted in a laboratory setting, where various stress factors like heat,^[^ ^20^ ^]^ humidity,^[^ ^21^ ^]^ and UV light^[^ ^22^ ^]^ are applied; alternatives such as TPO^[^ ^23^ ^]^ and POE^[^ ^24^ ^]^ have demonstrated superior optical, physical, chemical,^[^ ^25^ ^]^ mechanical, and PID^[^ ^26^ ^]^ performance compared to EVA. Further investigation extending from laminates^[^ ^27^ ^]^ or glass/backsheet constructions as considered in this study to glass/transparent, glass/glass^[^ ^28^ ^]^ and bifacial^[^ ^29^ ^]^ (for a review on degradation in bifacial modules, cf.^[^ ^30^ ^]^) constructions confirms the improvement potential underlying in devised encapsulation alternatives. The drive for materials that offer lower levelized cost of energy,^[^ ^31^ ^]^ extended service lifetimes, and are recyclable is therefore steering the selection toward stable, non‐crosslinked encapsulants for environmental concerns.

Environmental impacts of electricity generated by PV modules are influenced by a range of factors that span the entire life cycle of the PV modules, from the extraction of raw materials to manufacturing, transportation, installation, operation, end‐of‐life treatment, including waste management and recycling, along with service lifetime, performance, and degradation metrics.^[^ ^32^ ^]^ The production of PV modules is energy‐intensive, primarily due to the extraction and refinement of raw materials like silicon, as well as the manufacturing process of the cells.^[^ ^33^ ^]^ Consequently, a significant portion of recent Life Cycle Assessment (LCA) research on PV energy has concentrated on exploring the environmental impacts of various manufacturing methods;^[^ ^34^ ^]^ examining the effects of different life cycle stages;^[^ ^35^ ^]^ investigating PV technologies such as crystalline silicon,^[^ ^36^ ^]^ cadmium telluride (CdTe),^[^ ^37^ ^]^ passivated emitter and rear cell variants of crystalline silicon,^[^ ^38^ ^]^ perovskite,^[^ ^39^ ^]^ perovskite tandem^[^ ^40^ ^]^ and bifacial^[^ ^41^ ^]^ modules; and comparing technologies such as multi‐crystalline versus CdTe^[^ ^42^ ^]^ or between third‐generation photovoltaics.^[^ ^43^ ^]^ Many studies consider LCA of specific plant scales and system compositions in analysis and comparisons, for instance, small scale 1.8 kWp with batteries,^[^ ^44^ ^]^ distributed 2.7 kWp,^[^ ^45^ ^]^ standalone 4.2 kWp,^[^ ^46^ ^]^ large scale 100 MWp,^[^ ^47^ ^]^ very large scale 1 GWp;^[^ ^48^ ^]^ residential systems at low irradiation regions;^[^ ^49^ ^]^ more complex and special applications such as a building integrated window PV system,^[^ ^50^ ^]^ a floating system^[^ ^51^ ^]^ and an electric vehicle integrated system.^[^ ^52^ ^]^ Several other studies extend LCA with parametric analysis,^[^ ^53^ ^]^ integrating cost analysis^[^ ^54^ ^]^ or technical performance and economical feasibility analysis.^[^ ^55^ ^]^ The advantages of end‐of‐life management practices like recycling^[^ ^56^ ^]^ or using recycled material in modules^[^ ^57^ ^]^ is another extension in PV LCA. This has been considered with economic,^[^ ^58^ ^]^ feasibility,^[^ ^59^ ^]^ technological^[^ ^60^ ^]^ aspects and under circular economy^[^ ^61^ ^]^ and supply chain^[^ ^62^ ^]^ perspectives. Additionally, many studies have focused on PV systems situated in specific geographical regions and locations such as Australia,^[^ ^63^ ^]^ Singapore,^[^ ^64^ ^]^ China,^[^ ^65^ ^]^ Asia Pacific,^[^ ^66^ ^]^ Nigeria,^[^ ^67^ ^]^ North East England,^[^ ^68^ ^]^ Spain,^[^ ^69^ ^]^ USA,^[^ ^70^ ^]^ New York State/USA^[^ ^71^ ^]^ and Mexico.^[^ ^72^ ^]^ These studies assess contributions to the local electricity mixes and associated life cycle impacts. The research also covers how operational conditions and types of installations, such as fixed, tracked, concentrated, and rooftop affect environmental outcomes.^[^ ^73^ ^]^ Reviews on PV LCA covering the environmental impacts, energy requirements, and greenhouse gas (GHG) emissions of various PV systems, including amorphous, mono‐crystalline, multi‐crystalline, and advanced technologies,^[^ ^74^ ^]^ as well as CdTe and copper indium gallium selenide modules,^[^ ^75^ ^]^ are available. More recent reviews highlight grid‐connected PV systems, from first‐generation silicon‐based to third‐generation non‐silicon‐based technologies,^[^ ^76^ ^]^ and include thin film, dye‐sensitized, perovskite, and quantum dot‐sensitized solar cells,^[^ ^77^ ^]^ focusing on metrics like cumulative energy demand, energy payback time, and GHG emissions. Lastly, there is an emphasis on harmonizing these findings for broader applicability and understanding because the reported results often vary depending on the assumptions made and the size and technology of the systems under consideration. Harmonization is undertaken via approaches such as scenario comparison,^[^ ^78^ ^]^ systematic review,^[^ ^79^ ^]^ where some studies restrict within mono‐ and multi‐cystalline PV,^[^ ^80^ ^]^ emerging PV technologies,^[^ ^81^ ^]^ while others consider electricity generation from all renewables,^[^ ^82^ ^]^ or compile LCA studies that contribute to assessment of impacts with respect to time and geographical site.^[^ ^83^ ^]^ Despite this extensive coverage, there is a noticeable lack of comprehensive understanding regarding the advantages of extending the service lifetime of PV modules. With the shift in the industry shift toward offering power performance warranties extending from 25 to 30 years, Task 12 of the International Energy Agency Photovoltaic Power Systems Programme (IEA PVPS) has recently included a sensitivity analysis in its report.^[^ ^5^ ^]^ This analysis explored the environmental impact of prolonging the service lifetime of PV systems by an additional five years. The findings revealed that although extending the service lifetime increases maintenance and operational burdens, the resultant boost in electricity generation contributes to a 16% reduction in nearly all life cycle impact categories. Notably, the environmental benefits of extending the service lifetime of PV modules through alternative lamination processes that eliminate the issues related to EVA have scarcely been explored. This oversight presents a critical research opportunity as extending service lifetime directly influences the life cycle environmental impacts of PV energy by reducing the frequency of manufacturing, installation, and disposal processes. This study seeks to bridge this gap by assessing the potential environmental benefits of extending the service lifetime of PV modules beyond the current standard by conducting a comprehensive LCA that compares the two, thereby offering valuable insights into the sustainability advantages of increased service lifetime. By doing so, this study aims to provide a comprehensive evaluation of the consequent reductions in environmental impacts, offering an important perspective on how module service lifetime can serve as a pivotal factor in the sustainability of PV energy systems. This approach not only diverges from the traditional focus of existing research but also introduces an essential dimension to the discourse on sustainable energy solutions, underlining the imperative of module service lifetime in minimizing the environmental impacts of PV technology.

Given that current power performance warranties offer a service lifetime of 30 years, the adoption of improved lamination techniques is assumed to extend this lifetime to 40 years. When modeling an alternative lamination process, possible changes in material and energy usages, as well as the maintenance inputs considering the extended service lifetime, were deliberated in this study. Additionally, the improvement in performance during the extended service lifetime was estimated. Moreover, the variability that would possibly emerge in the levels of process input requirements at various stages of an alternative process was also accounted for. To comprehensively understand the environmental impacts, the LCA analysis in this work was designed under various scenarios that include a selection of parameter settings that adequately represent the level of uncertainty. These scenarios were modeled such that the effects of altering encapsulation material, energy consumption requirements needed for lamination, and maintenance needs in the extended service lifetime, were effectively demonstrated. In this way, evaluating the environmental impact of each scenario enabled the identification of the most sustainable solutions that strike an optimal balance between performance and resource efficiency.

This study stands at the forefront of addressing a critical yet underexplored dimension of PV energy sustainability. By pioneering a comprehensive analysis of the environmental impacts associated with extended service lifetimes of PV modules, this study promises to deliver valuable insights. These insights will not only challenge current manufacturing and recycling paradigms but also provide a robust empirical foundation for future policy and investment decisions in the renewable energy sector. Through this work, it is aimed to catalyze a paradigm shift toward longer‐lasting, more sustainable PV systems, significantly reducing the environmental impacts of PV energy generation. Ultimately, findings in this study are poised to guide the renewable energy community in pursuing strategies that enhance the sustainability and economic viability of PV systems, making a substantial contribution to the global transition toward clean energy.

The organization of the paper is as follows. Section 2 presents and discusses the results of the LCA analysis for the 40‐year systems, evaluating different scenarios for the extended service lifetime. This includes a comparative analysis of the environmental impacts between systems with 30‐year and 40‐year service lifetimes, alongside a Monte Carlo simulation to evaluate uncertainty. Section 3 offers concluding observations on the environmental impact studies, assessing different scenarios to aid decision‐making, and provides suggestions for the manufacturers. Section 4 provides an overview of the methodologies employed, detailing the assumptions, choices, and analytical methods applied in the LCA and sensitivity analyses. Detailed analyses of the LCA for 30‐year systems, including impact comparisons between multi‐crystalline and mono‐crystalline modules and the influence of end‐of‐life treatment through module recycling, are available in the Supporting Information. This section also contrasts modules using EVA with those employing alternative encapsulating materials.

## 2 Results and Discussion

### 2.1 LCI for 40‐Year PV Power Plants

Since the extension of the service lifetime from 30 to 40 years implies a potential increase of electricity generation, the environmental impact of the initial commissioning processes can be significantly reduced when evaluated per unit of electricity generated. However, it is possible that there might be a partial increase in environmental burdens due to the new lamination process and changes in the requirements associated with extending the plant's service lifetime. This increase could be attributed to higher energy consumption during module manufacturing and the needs for repair and replacement of modules, inverters, cabling, and mounting system components throughout the extended service lifetime. The balance between increased input requirements and energy output was examined here through four different scenarios: optimistic, moderate, pessimistic, and pessimistic‐efficient.

#### 2.1.1 Scenario‐based Updates on PV Module LCIs due to Process Innovations in Manufacturing

With the exception of materials requiring higher temperatures for the lamination, such as ionomers, each lamination process for alternative materials, such as POE and TPO, does not necessitate higher temperature or pressure requirements than the current standard EVA processing. The specific temperature and pressure regime required for lamination with EVA is as follows: applying a vacuum of 0.1 bar to evacuate air pockets within the laminated structure for 5 min while pre‐heating from ambient temperature to ≈60–80 °C with a heating rate of ≈5–10 °C min^−1^ for EVA to soften, flow, and embed the cells, followed by raising the temperature to ≈150 °C to facilitate crosslinking reactions in the polymer in this curing stage under a pressure of 1 bar for 10 min. As a result of this process, the EVA acquires elastomeric, rubber‐like properties with a gel content of ≈70 to 80%.

The pessimistic scenario, which encompasses processes with higher lamination temperatures, pressures, or longer application times, assumes a 10% increase in electricity and diesel consumption in energy demand in the overall inventory of module production and installation processes. The optimistic scenario, which considers no changes in the processing conditions, assumes no increase in energy demand due to lamination and related processes. The moderate scenario is in between the two scenarios, and thus, considers a 5% increase.

The potential changes in the environmental impacts due to modifications in the lamination polymer were explored by comparing modules with EVA and with an alternative polymer. Since encapsulants based on polyethylene copolymers such as POE and TPO considered as alternatives to EVA do not have defined flows in the ecoinvent database, PET polymer was selected instead as a proxy material due to data quality concerns, and the LCA analysis was conducted with this input. In this case, it was determined that the changes in the environmental impacts were negligible with the use of different polymeric material than EVA as shown in Table S5 (Supporting Information) and Figure S3 (Supporting Information). Therefore, when different scenarios, including energy requirements for the maintenance and repair of system components after module production and installation, were considered, EVA was kept constant as the encapsulant material used for module lamination.

#### 2.1.2 Scenario‐Based Updates on PV Plant LCIs due to Balance‐of‐System Components During the Extended Service Lifetime

Regarding the mounting infrastructure required for installation, it is composed of durable construction materials with a service lifetime of 60 years.^[^ ^84^ ^]^ Although there might be occasional repairs or replacements, the likelihood is very small compared to other failures or can be considered negligible. Hence, the optimistic scenario assumes no increase in the mounting systems input during the service lifetime of 40 years with the possibility of using the same system for the additional 10 years. In the moderate and pessimistic scenarios, a 5% and a 10% increase, are assumed, respectively, to account for potential replacements or repairs.

The service lifetime of inverters is assumed to be 15 years, but they can significantly contribute to the overall failure rate in a relatively short period.^[^ ^84^ ^]^ Depending on the capacity of inverters, they are usually constructed using large housings, allowing easy access and maintenance. By adding 10% in weight as additional repair and maintenance burden for every 10 years, inverters can continue to function. This view forms the basis of the optimistic scenario in this study. It is therefore assumed that with regular maintenance and repair, the two 15‐year inverters would function with an additional 10% burden, enabling the inverters to operate until the end of 40 years. In the pessimistic scenario, adhering to the service lifetime of 15 years and assuming a linear 33% increase in the inverter inventory for the additional 10 years, the inverters need to be replaced again after 30 years. Here, a 33% increase in load is considered instead of 50%, considering the use of the lastly installed inverter possibly in another application outside the boundary of this analysis for the rest of its lifetime.

For cabling, the situation is critical. The service lifetime for cabling is assumed to be 30 years, and at the end of 30 years, the cables are either thoroughly inspected and partially replaced or a completely new electrical installation is made. However, with careful periodic maintenance, rigorous testing, control, and repairs, cables can be significantly preserved and maintained in service.^[^ ^84^ ^]^ Therefore, in the optimistic scenario, it is assumed that 30% of complete cabling is needed to sustain the system for the additional 10 years, considering the required maintenance and repairs. In the pessimistic scenario, an additional 90% burden is assumed to become necessary after 30 years for almost a new installation, excluding the maintenance and repairs for 20 years that are outside the analysis boundary.

An effective innovative lamination and module production process can ensure efficient operation during the last 10 years without imposing additional burden for module repairs and replacements. Accordingly, in the optimistic scenario, the module repair and replacement rate of 2% is assumed as stated as the base case in the life cycle inventory. In the pessimistic scenario, due to the use of aged modules during the last 10 years, an additional 2% is assumed, summing up to a total module repair and replacement rate of 4% over the service lifetime of 40 years to account for unforeseen effects that may be observed in modules during the extended lifetime and to consider potential life cycle impacts.

The electricity, diesel, and transportation inputs required to commission the power plant were adjusted based on the changes in mass inputs due to the different scenarios. This approach is consistent with the inventory information provided in Section S1 (Supporting Information) for the power plant with a service lifetime of 30 years.

In addition to optimistic, moderate, and pessimistic scenarios, an efficient pessimistic scenario was also evaluated. In this scenario, (i) degradation/output performance and module repairs are kept at a level similar to the optimistic scenario, (ii) the input increase for the laminating process, covering material and process alternatives realistically, is in line with the moderate scenario, and (iii) other power plant component parameters are adapted from the pessimistic scenario. Here, a 5% increase in additional energy demand due to electricity and diesel requirements of the module production process is assumed to be reasonable. **Table** **1** summarizes the parameter settings for these scenarios. The effect of differences between these scenarios on the impact categories will be explored in detail by Monte Carlo simulation and subsequent pairwise Tukey tests, along with Cohen's D effect sizes, in Section 3.3.

**Table 1:** Increases in inputs (%) according to optimistic, moderate, pessimistic, and pessimistic‐efficient scenarios when the service lifetime is extended from 30 to 40 years.

	Optimistic	Moderate	Pessimistic	Pessimistic‐Efficient
Electricity (module production)	0	5	10	5
Diesel (module production)	0	5	10	5
Module repairs	0	50	100	0
Inverter	10	20	33	33
Mounting systems	0	5	10	10
Electric installation	30	60	90	90

#### 2.1.3 Scenario‐Based Updates on Electricity Generation LCI due to Degradation in Output Performance During the Extended Service Lifetime

**Figure 1:**
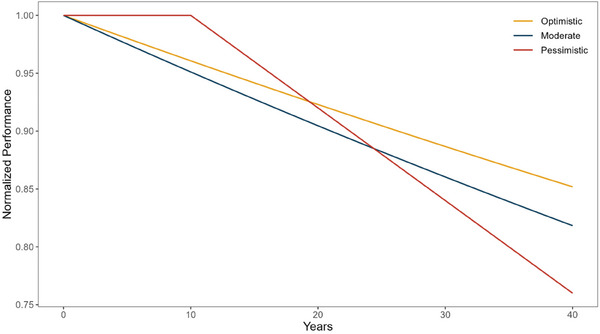
Module performance degradation curves for optimistic, moderate, and pessimistic scenarios for PV power plants with a service lifetime of 40 years.

While extending the lifetime from 30 to 40 years, performance losses have to be considered in order to estimate the total electricity output of the PV modules or systems throughout the service. This was assessed by using degradation rates obtained from field results.^[^ ^85^ ^]^ The optimistic scenario assumes that the improvements lead to a service lifetime of 40 years with an average module performance spread over 40 years, resulting in a linear degradation rate of ≈0.35% per year. Consequently, the modules in the optimistic scenario continue to generate at ≈85% of their initial performance after 40 years. Conversely, the pessimistic scenario assumes that the module performance remains stable for the first 10 years and then degrades linearly at a relatively higher degradation rate of ≈0.6% per year. In this case, the performance ratio at the end 40 years is ≈75% of the initial performance, which fails to meet the current performance warranty of 80%. The moderate scenario assumes a linear degradation rate of around 0.525% per year, indicating faster degradation in the initial stage, which gradually decreases over the years, with a performance ratio of ≈80%. This results in a higher proportion of output achieved in the last 10 years compared to the pessimistic scenario. The total electricity generation of a power plant with a service lifetime of 40 years, compared to that with 30 years, is estimated to be 133.3%, 130%, and 128.2% for the optimistic, moderate, and pessimistic scenarios, respectively. Degradation curves studied and their effects on the performance over years can be seen in **Figure** **1**.

### 2.2 Analysis of the Deterministic LCIA Results for 40‐Year PV Power Plants

After constructing LCIs that include process innovations in module manufacturing, adjustments for the burdens associated with balance‐of‐system components in plant commissioning, and estimations for the electricity generation due to performance and degradation, during the extended service lifetime, deterministic life cycle impact results can be calculated under scenarios that reflect the range of uncertainty involved in LCI updates.

**Table** **2** reports the environmental impact analysis results of 1 kWh electricity generation from the 570 kWp PV power plant with a service lifetime of 40 years that uses modules produced with the improved lamination process under different scenarios, along with the results from the same plant with the current standard service lifetime of 30 years. It is to be noted that the abbreviations used for impact categories can be found in Section S2 (Supporting Information). By comparing the effects of these scenarios, this study aims to inform decision‐making processes, demonstrate the sensitivity of impacts to increasing resource use, and identify areas that require improvements to reduce environmental burdens.

**Table 2:** Environmental impacts of 1 kWh electricity generation in terms of ReCiPe midpoint impact categories from the 570 kWp PV power plants with a service lifetime of 30 years and 40 years under optimistic, moderate, pessimistic, and pessimistic‐efficient scenarios.

Impact Category	30‐Year	40‐Year Optimistic	40‐Year Moderate	40‐Year Pessimistic	40‐Year Pessimistic‐Efficient	Unit
FMFP	8.77E‐05	6.05E‐05	6.40E‐05	6.70E‐05	6.33E‐05	kg PM2.5‐eq
FFP	1.05E‐02	7.67E‐03	8.06E‐03	8.38E‐03	7.95E‐03	kg oil‐eq
FETP	8.41E‐03	6.96E‐03	8.07E‐03	9.16E‐03	8.79E‐03	kg 1,4DCB‐eq
FEP	2.16E‐05	1.62E‐05	1.73E‐05	1.83E‐05	1.74E‐05	kg P‐eq
GWP	3.75E‐02	2.72E‐02	2.86E‐02	2.97E‐02	2.82E‐02	kg CO_2_‐eq
HTPc	6.29E‐01	4.53E‐01	4.86E‐01	5.17E‐01	4.95E‐01	kg 1,4DCB‐eq
HTPnc	2.05E+01	1.45E+01	1.60E+01	1.75E+01	1.67E+01	kg 1,4DCB‐eq
IRP	4.84E‐03	3.38E‐03	3.54E‐03	3.67E‐03	3.48E‐03	kBq Co‐60‐eq
LOP	1.25E‐02	9.39E‐03	1.01E‐02	1.07E‐02	1.03E‐02	m^2^·yr
METP	2.63E+01	1.92E+01	2.13E+01	2.33E+01	2.23E+01	kg 1,4DCB‐eq
MEP	3.07E‐06	2.10E‐06	2.20E‐06	2.28E‐06	2.15E‐06	kg N‐eq
SOP	7.80E‐04	2.60E‐04	3.00E‐04	3.30E‐04	3.20E‐04	kg Cu‐eq
HOFP	1.10E‐04	7.77E‐05	8.20E‐05	8.56E‐05	8.13E‐05	kg NO_x_‐eq
EOFP	1.10E‐04	8.08E‐05	8.53E‐05	8.91E‐05	8.46E‐05	kg NO_x_‐eq
ODP	2.45E‐08	1.78E‐08	1.88E‐08	1.96E‐08	1.86E‐08	kg CFC‐11‐eq
TAP	1.60E‐04	9.86E‐05	1.00E‐04	1.10E‐04	1.00E‐04	kg SO_2_‐eq
TETP	6.83E‐01	3.23E‐01	3.38E‐01	3.51E‐01	3.32E‐01	kg 1,4DCB‐eq
WCP	1.00E‐03	6.80E‐04	7.10E‐04	7.40E‐04	7.00E‐04	m^3^

**Figure 2:**
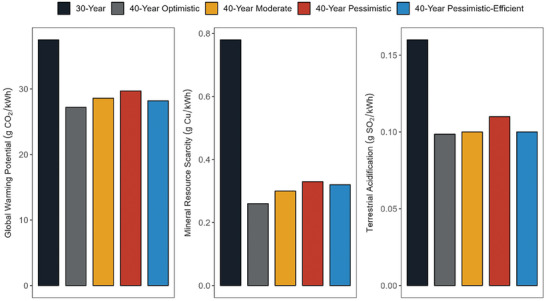
Comparison of environmental impacts of 1 kWh electricity generation in terms of global warming potential, mineral resource scarcity, and terrestrial acidification categories from the 570 kWp PV power plants with a service lifetime of 30 years and 40 years under optimistic, moderate, pessimistic, and pessimistic‐efficient scenarios.

**Figure** **2** depicts the global warming potential, mineral resource scarcity, and terrestrial acidification categories because these categories are particularly crucial in the life cycle of PV systems. All the 40‐year scenarios demonstrate marked improvements compared to the 30‐year scenario. For global warming potential, the optimistic scenario shows the most significant reduction, with emissions lowering to 27.2 g CO_2_‐eq from 37.5 g CO_2_‐eq. It is followed closely by the pessimistic‐efficient scenario, which records emissions at 28.2 g CO_2_‐eq, and the moderate scenario at 28.6 g CO_2_‐eq. The pessimistic scenario, while still better than the 30‐year scenario, shows the highest emissions among the 40‐year scenarios at 29.7 g CO_2_‐eq. Both the moderate and pessimistic‐efficient scenarios suggest a pragmatic, albeit less aggressive, approach to emission reductions than the optimistic scenario, balancing environmental concern with practical feasibility. The situation is more pronounced in mineral resource scarcity. This is largely because key components such as cell metallization, electrical cabling and wiring, module frames, and mounting systems for module installations rely heavily on metal usage. By extending the service lifetime, the demand for fresh mineral resources can be significantly reduced, mitigating the environmental impacts associated with their extraction and processing.

The findings from the analysis indicate significant variations in environmental impacts across different scenarios. The optimistic scenario stands out with markedly lower impacts in several key categories compared to the other scenarios. Notably, reductions in mineral resource scarcity, terrestrial ecotoxicity, and terrestrial acidification are observed at 67%, 53%, and 38%, respectively, when compared to the plant with a service lifetime of 30 years. Other environmental impact categories see reductions ranging between 25% and 32%, except for freshwater ecotoxicity, which shows a comparatively modest reduction of 17%. These improvements are largely attributed to advances in the lamination process, which enhance the durability and reliability of module structures during outdoor deployment, alongside better maintenance and repair practices. Collectively, these developments in the optimistic scenario contribute to a significant mitigation of adverse effects on human health, the environment, and resource depletion across multiple midpoint categories.

Conversely, the pessimistic scenario yields smaller reductions, between 11% and 26% across most categories, but it records a 9% increase in freshwater ecotoxicity. Sensitive categories such as marine ecotoxicity, freshwater ecotoxicity, and human non‐carcinogenic toxicity show at least 15% more impacts under this scenario compared to the optimistic scenario.

The pessimistic‐efficient scenario offers some recovery of impacts, typically ranging from 3% to 9% improvement over the pessimistic scenario. Nonetheless, reductions of 20% or more remain typical when compared to 30‐year scenario. Successful realization of module and system improvements in this scenario could lead to considerable environmental benefits, even as other power plant components face increased maintenance challenges over the extended service lifetime of 40 years.

Global warming potential sees its most substantial improvement in the optimistic scenario with a 27% reduction compared to the 30‐year baseline. The pessimistic‐efficient, moderate, and pessimistic scenarios follow with reductions of 25%, 24%, and 21%, respectively. Notably, the pessimistic‐efficient scenario occasionally outperforms or matches the moderate scenario across most impact categories.

**Figure 3:**
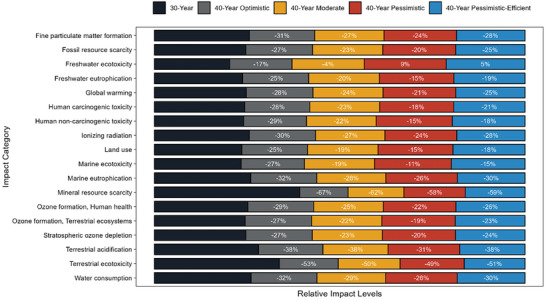
Comparison of environmental impacts of 1 kWh electricity generation in terms of ReCiPe midpoint impact categories from the 570 kWp PV power plants with a service lifetime of 30 years and 40 years under optimistic, moderate, pessimistic, and pessimistic‐efficient scenarios.

These results highlight the environmental benefits of extending the service lifetime of modules and systems from 30 to 40 years, particularly with enhancements in the lamination process. This extension appears to be beneficial across nearly all impact categories, even under conditions demanding frequent maintenance and repairs. These comparative advantages are visually depicted in **Figure** **3**, emphasizing the impact reductions (%) achieved by extending the service lifetime from 30 to 40 years under the scenarios examined.

### 2.3 Statistical Analysis of the Monte Carlo Simulation Results

Figure 3 presents the deterministic impact values, calculated by assigning median values from the data sources to all inputs and outputs in the life cycle inventories. To address the inherent variability and uncertainty in these median values, Monte Carlo simulations were carried out for each scenario with a sample size of 60, as detailed in Section 2.4. Each of the samples contain impact results in 18 categories, and since comparisons and hypothesis tests are conducted within categories, no data preprocessing, normalization or transformation is necessary for sample impact results, and no samples are left out as outliers for any scenario or impact category. All statistical analyses are conducted using MATLAB 2022a Statistics Toolbox. Sample mean and standard deviations for each scenario and impact category are available in Table S6 (Supporting Information). Table S7 (Supporting Information) lists the (one‐sided) p‐values from one‐way ANOVA tests for each ReCiPe Midpoint category under the 30‐year and 40‐year scenarios. These results clearly indicate that scenario has a significant effect on environmental impacts across all categories. The Tukey pairwise comparisons revealed significant differences between the impacts of the 40‐year scenarios and the 30‐year scenario. Given that all (two‐sided) p‐values were equal to 0, these specific results are omitted from the table for brevity. Furthermore, effect sizes were calculated and presented as Cohen's D values in Table S8 (Supporting Information). The analysis shows that the 40‐year scenarios, especially the pessimistic one, consistently resulted in significant reductions in impact across most categories when compared to the 30‐year scenario. The effect sizes were notably large in nearly all categories, with the largest and smallest effect sizes being 52.5 and 1.31, respectively. These substantial effect sizes further reinforce the significant differences identified in the Tukey test results.

**Figure 4:**
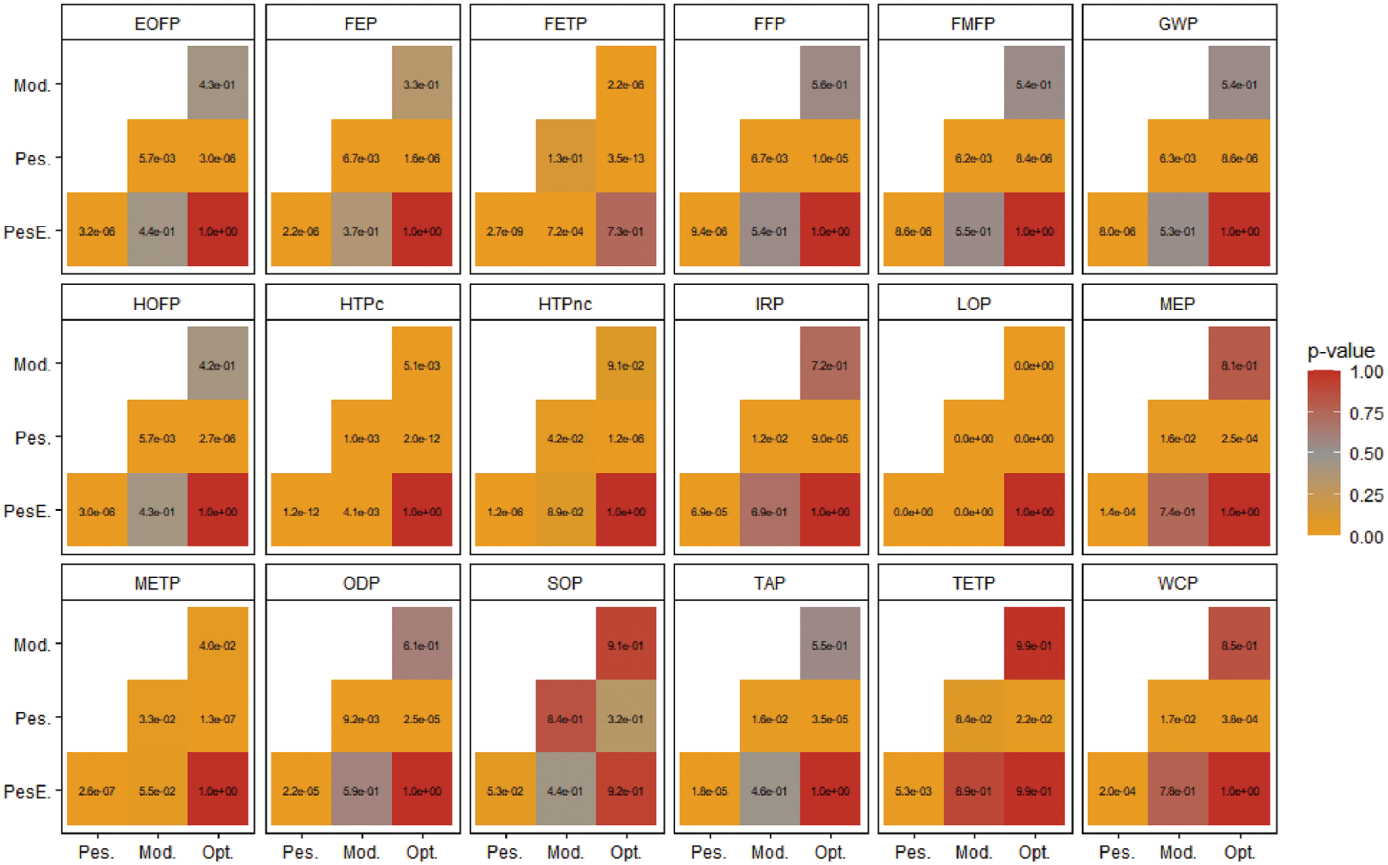
The results of Tukey pairwise scenario comparisons with p‐values (subsequently performed after one‐way ANOVA test) for the ReCiPe Midpoint environmental impact categories on Monte Carlo samples (n = 60) generated for the 570 kWp PV power plants with a service lifetime 40 years under different scenarios.

The heatmap in **Figure** **4** focuses exclusively on the pairwise comparisons between the 40‐year scenarios. The corresponding effect sizes are detailed in Table S9 (Supporting Information) and Figure S5 (Supporting Information). Statistically significant differences, indicated by *p*‐values less than 0.05, are observed between the following scenario pairs: optimistic versus pessimistic, moderate versus pessimistic, and pessimistic versus pessimistic‐efficient. The relatively larger effect sizes indicate substantial differences in impact levels across most categories between these scenario pairs. This suggests that the variations between the pessimistic and other scenarios are not only statistically significant, but also practically significant and impactful.

The comparison between the pessimistic versus pessimistic‐efficient scenario pair is particularly noteworthy. The pessimistic‐efficient scenario shows significantly lower impacts, highlighting the vital role of module development and the advantages of sustaining efficient module performance throughout the extended service lifetime. This effectively surpasses the impact of all other system components in terms of environmental benefits.

Conversely, the optimistic versus pessimistic‐efficient scenario pair shows no statistically significant differences across any impact categories, with p‐values substantially above 0.05. The small effect sizes observed here indicate that any existing differences between these scenarios are likely negligible in practical terms. This supports the hypothesis that the benefits derived from extending the service lifetime of modules outweigh the additional burdens imposed by balance of system components such as mounting systems, cables, and inverters.

For the optimistic versus moderate and moderate versus pessimistic‐efficient scenario pairs, the majority of impact categories show no statistically significant differences, with p‐values exceeding 0.05, except in toxicity‐related categories (e.g., freshwater and marine ecotoxicity, human carcinogenic and non‐carcinogenic toxicity). Here too, the modest effect sizes suggest that any existing differences are not large enough to be of practical relevance. It appears that both the service life output performance and additional burdens under the moderate scenario are closely aligned with those of the optimistic scenario. This leads to a situation where moderate increases in performance effectively offset any moderate increases in inventory burdens, and thus, the life cycle impacts are comparable and not significantly more adverse than those observed in the optimistic or pessimistic‐efficient scenarios.

In summary, the Monte Carlo simulations and subsequent statistical analysis have confirmed the positive environmental impacts of extending the service lifetime of modules from 30 to 40 years. The analysis reveals that, in terms of statistical significance, the optimistic and pessimistic‐efficient scenarios perform notably better than other 40‐year scenarios. Enhancements to PV modules that enable extended service lifetimes significantly mitigate the impacts of potential burdens during the additional 10 years of plant operation. While the pessimistic scenario displays significant differences and large effect sizes in comparison to other scenarios, the optimistic and moderate scenarios generally do not, and their effect sizes remain modest. Thus, moderate deviations from performance targets of the PV module enhancements and expectations of efficient plant maintenance in the extended service lifetime are considered manageable.

## 3 Conclusions

In this work, the effect of extending the service lifetime of PV modules from the standard 30 years to 40 years on environmental impacts was investigated using life cycle assessment. The findings indicated substantial environmental benefits, especially in crucial categories such as global warming potential and mineral resource scarcity. These benefits persisted across a variety of scenarios that considered different rates of module performance degradation and varying behaviors of balance‐of‐system components over the extended service lifetime. This held true even when taking into account the uncertainty of life cycle inventory parameters. Both optimistic and pessimistic‐efficient scenarios demonstrated measurable and significant environmental improvements attributable to enhancements in PV modules. Additionally, further environmental impact reductions could be achieved through improvements in balance‐of‐system components. These components were particularly influential in moderate and pessimistic scenarios.

To significantly extend the service lifetime and enhance the durability of PV modules, manufacturers should consider adopting advanced encapsulant materials such as POE, TPO, and ionomers, which offer better resistance to degradation, compared to traditional EVA. A rigorous quality control process, including optimal curing parameters and routine integrity checks using non‐destructive methods, is crucial in the lamination process. Innovations in layering techniques, such as optimizing the thickness and uniformity of layers and adding better stabilizers to encapsulant formulations, can further enhance humidity, UV resistance, and mechanical stability. Sustainable manufacturing practices, including minimizing material waste and enhancing energy efficiency in heating and cooling cycles during lamination, not only improve production efficiency but also contribute to environmental sustainability. Collaborating with material scientists and suppliers to develop tailored encapsulant formulations and engaging in accelerated indoor testing and field testing under real‐world conditions can provide valuable insights for continuous improvement. Additionally, focusing on the end‐of‐life stage by designing for recyclability for efficient module recycling will help recover valuable materials and reduce environmental impacts. For example, TPO and ionomers, both recyclable materials, enhance the overall recyclability of module designs. Additionally, the adoption of double glass construction eliminates the need for non‐recyclable fluoropolymer‐based backsheets, further boosting recyclability and making them more environmentally friendly. While these strategies may involve higher upfront costs, these would be balanced by the extended service lifetime of modules, ultimately leading to a reduction in the levelized cost of PV electricity and rendering it a cost‐effective alternative to traditional modules with EVA encapsulants. With the adoption of more sustainable production methods, such as the improved lamination process examined in this study, manufacturers can achieve significant advancements in module performance and sustainability, and the production and energy industries can move toward a greener future and ultimately contribute to global sustainability efforts.

## 4 Experimental Section

### Goals and Scope

In this study, a cradle‐to‐grave LCA was considered for a 570 kWp open‐ground PV power plant. The functional unit of the power plant was defined as 1 kWh of electricity generated. The aim is to examine the environmental impacts associated with each stage of the plant's life cycle and to identify the effects of an alternative lamination process for PV module production on the impacts; therefore, the decision‐making context in the LCA stage was decided as Scenario A.^[^ ^86^ ^]^ The system boundaries encompass module production, plant construction, assembly, electricity generation, plant maintenance, and end‐of‐life treatments (where applicable).

This study primarily addresses glass/backsheet type modules; however, it is important to acknowledge the burgeoning research surrounding glass/glass modules. The choice of encapsulant is particularly critical in these modules due to the impermeability of glass, which leads to a sealed environment. Utilizing EVA in this context can result in significant issues with the formation of acidic acid upon degradation; when trapped within the glass/glass module, this can accelerate corrosion of the cell metallization and lead to severe discoloration.^[^ ^87^ ^]^

### LCI

Relevant databases and literature sources, including ecoinvent^[^ ^88^ ^]^ and the IEA PVPS's LCA reports, comparing earlier LCIs^[^ ^89^ ^]^ with later versions^[^ ^90^ ^]^ and recent updates,^[^ ^32^ ^]^ were utilized to gather the LCI data. Since the main objective of this study is to compare alternative options, an economic allocation method was chosen to address multi‐functional processes in the background, assuming that there were no multi‐functional processes in the foreground. Thus, an LCI modeling framework based on average processes through economic allocation was adopted in the initial deterministic analyses. Moreover, regional providers were identified as Turkey (TR) for the cell and module production, and Europe‐other (RER) and global (GLO) markets for other inventory contents.

The life cycle inventory for the production of mono‐crystalline PV modules, which is one of the main foreground processes in this study, was adapted from the inventory in the IEA PVPS's LCA report. Since the production processes for alternative lamination polymers such as POE or TPO are not available in the ecoinvent database, polyethylene terephthalate (PET) polymer was chosen as a proxy material to represent the production processes of these alternative polymers. The choice of PET particularly depends on data quality concerns since this material has a defined process in the database.

To construct an up‐to‐date life cycle inventory for the mono‐crystalline PV modules, several inventories were analyzed and compared. The more recent inventory in the of the IEA PVPS's LCA report is more intensive in terms of water and energy usage while being more economical in terms of other material inputs such as glass and encapsulant. The inventories for the current standard EVA module and the module with an alternative encapsulant material are presented in Table S1 (Supporting Information). After an analysis of the changes from previous inventories to the more recent one, a modification of upstream processes that reflects up‐to‐date module technology, performance, and applications was performed.

Currently, mono‐crystalline modules hold approximately 85% of the market share with module efficiency values of 20% and above.^[^ ^91^ ^]^ In this study, an average of 200 W_p_ m^−2^ was therefore considered as the current standard for module peak performance. Consequently, for a 570kWp power plant, 2940 m^2^ of modules were supplied, considering the 3% repair and replacement ratio, 2850 m^2^ of which were assumed to be deployed and actively used. This value also corresponds to the amount of the mounting system input required for the installation and commissioning of the PV power plant.

Except for the inverter, the balance‐of‐system components such as electrical cabling/wiring and mounting infrastructure are directly proportional to the area of modules, so a mass adaptation was applied for the required transportation and fuel inputs. The inverter installation depends on the plant's sizing and an inverter with 500 kW capacity was used for this 570 kWp power plant installation. The assumed lifetime of the inverter is 15 years, and considering one replacement during the plant's service lifetime, the total weight used is calculated to be 9330 kg (4665 kg for 15 years).^[^ ^84^ ^]^ Considering 13.2 kg m^−2^ module weight, 12.8 kg m^−2^ mounting system weight, and 0.55 kg m^−2^ electrical wiring weight per installed module area, inverters constitute ≈10% of the total weight. Table S2 (Supporting Information) illustrates the required flows for the installation of the power plant, including the module mounting system components, which comprises the required materials and construction activities required for installation and commissioning.

Currently, modules are marketed with 80% performance warranty at the end of service lifetime of 30 years. The average annual performance value was taken as 1500 kWh per kWp per year for the power plant, assumed to be located in Central Anatolia, Turkey (latitude 40°N), which represents a wide range of global locations near middle latitudes with a moderate economic viability. Then, the 570 kWp power plant was estimated to produce a total of 25.65 GWh electricity during the entire service lifetime for 30 years. That is, one kWh of electricity produced requires 3.90E‐8 units of 570 kWp power plant. The primary maintenance operation for the PV power plant involves cleaning the modules once a year, in order to reduce the power losses due to soiling issues, requiring an average of 0.005 liters of water per kWh. Additionally, solar energy not converted to electrical energy is released into the atmosphere as heat and this was modeled as heat waste output in the amount of 0.25 MJ kWh^−1^. These flows required to operate the 570 kWp power plant are reported in Table S3 (Supporting Information).

### LCIA

The life cycle impact analysis (LCIA) was carried out using the OpenLCA (v1.11)^[^ ^92^ ^]^ software with the ReCiPe method on midpoint impact categories. The ReCiPe method addresses a range of environmental concerns at the midpoint level and aggregates them into three endpoint categories: human health, ecosystems, and resources.^[^ ^93^ ^]^ The focus was given on global warming potential, mineral resource scarcity, terrestrial acidification categories as these are particularly important in the life cycle of PV systems due to prominent environmental concerns and the amount of metal used in cell metallization, cabling/wiring for electrical installation, module frames, and mounting systems for module installation.

To assess the potential variations in environmental impacts under different scenarios, a sensitivity analysis was also conducted. The analysis was designed to examine the effects of changes in key parameters at the electricity generation, plant commissioning, and module production levels. Different scenarios related to the potential improvement were considered as optimistic, moderate, pessimistic, and pessimistic‐efficient. Details about these scenarios can be found in Section 3.1.

### Monte Carlo Simulation

Monte Carlo analysis applied to LCA involves a series of structured steps to assess uncertainty and variability, providing valuable statistical validation of scenario comparisons across impact categories. Key phases of this analysis are:

- Identifying sources of uncertainty in the LCI entries and determining probability models for each.
- Ensuring convergence of sample statistics by selecting an appropriate number of iterations for Monte Carlo simulation, balancing an informative sample while avoiding falsely significant results due to exceedingly large samples.
- Running Monte Carlo simulation: Conducting simulations for each iteration by sampling random variables for each uncertain LCI entry and calculating impacts for each life cycle category.
- Analyzing Monte Carlo impact results: Statistically analyzing impact results, considering sample statistics and necessary hypothesis tests.

Problem‐specific considerations include uncertainty modeling and interpretation of results, subsequently addressed for the scenarios considered in this study.

When generating life cycle inventories, data is usually gathered from experienced domain experts in relevant industrial processes and various databases containing information about products and processes. In this case, the quantities of input or output used for a unit reference process in life cycle inventory tables are provided based on a central tendency criterion known as a deterministic value. The central tendency criterion is typically the median or mean, and it is calculated based on the sample collected. The uncertainty inherent in the presented deterministic input and output quantities is usually supported by additional information in the tables that indicates the probability distribution of the relevant variable. In the ecoinvent tables, *SD95* data, representing 95% of the standard deviation for the log‐normal distribution, is commonly provided,^[^ ^88^ ^]^ as well as in the LCI sources supplementing the IEA PVPS LCA report.^[^ ^32^ ^]^ This model also applies to the inventories used in this study, where it is assumed that the table inputs follow a log‐normal distribution, and the given deterministic value (*M*) is the median of the distribution. The natural logarithm of this value (*µ*) represents both the median and the mean values of the log‐transformed data. The standard deviation (*σ*) of the log‐transformed data is calculated as follows:

(1) $$\mu =\ln\left(M\right)$$

(2) $$\sigma =\ln\left(\sqrt{SD95}\right)$$

The mean (ν) and standard deviation (γ) of the non‐transformed variable, i.e., the quantity of input or output per unit reference value, with a log‐normal distribution, are then calculated as follows:

(3) $$\nu =e^{\mu +\sigma^{2}/2}$$

(4) $$\gamma ={\left(\left(e^{\sigma^{2}}-1\right)e^{2\mu +\sigma^{2}}\right)}^{1/2}$$

Some LCA software, including the OpenLCA (v1.11) used in this study, take the geometric mean (*e* ^µ^) and geometric standard deviation (*e* ^σ^) as parameters for log‐normal distribution.

After selecting the uncertainty model ‐distribution and parameters‐ for all relevant inputs in the life cycle inventories, the uncertainty in LCIA results was calculated as confidence intervals based on Monte Carlo simulations. For each scenario, Monte Carlo simulations were used to generate samples, and then, the environmental impacts of different scenarios were compared by subjecting Monte Carlo samples to ANOVA and subsequent pairwise Tukey tests to determine whether the scenarios resulted in a significant difference in environmental impact categories. As a reminder, the significance level for p‐values is commonly set at 0.05, indicating that hypothesis tests yield significant results for p‐values below this threshold. An ANOVA test p‐value below 0.05 suggests that differences in an impact category among scenarios are significant. Similarly, in Tukey tests following ANOVA, a p‐value less than 0.05 indicates significant difference in impact category results between the compared scenario pair. p‐values alone might be misleading, particularly with large samples, thus they are supported by Cohen's D effect sizes. Cohen's D values lower than 0.2, around 0.5, and larger than 0.8 represent small, medium, and large effect sizes, respectively.

For a summary of the methods applied, Figure S4 (Supporting Information) illustrates the steps of the analysis workflow established in this study. The framework entails scenario‐based analysis across a hierarchy including LCIs of electricity generation from the 570 kWp PV power plant, the plant itself, and the PV modules installed in the plant. The LCI of electricity generation is determined by the reciprocal of the total lifetime output from the plant, influenced by factors such as the rated output, the plant's operational years, and its average annual output yield. The average yield is contingent upon module output degradation over the service lifetime, which is explored under various scenarios to capture the uncertainty in the extended service lifetime. The plant serves as an input to the electricity generation LCI, with its own updates accounting for increased burdens in components like inverters, mounting systems, and electric cabling. Modules are pivotal inputs in the PV plant LCI, making significant contributions across most impact categories. Proposed enhancements in PV modules are scrutinized, considering heightened burdens in energy and material inputs during module manufacturing.

Subsequent to these analyses and the formation of LCIs within the hierarchy, the study proceeds with the selection of LCA impact calculation methods and parameter choices, followed by the analysis and interpretation of impact results, mainly focused on comparing scenario alternatives and different service lifetimes across 18 impact categories. Monte Carlo analysis necessitates additional steps including data collection for LCIs, computation of sample impact results, and statistical analysis. Data analysis and visualization for comparing scenario alternatives, including statistical analysis of Monte Carlo samples using ANOVA and Tukey's pairwise tests, are followed by interpreting the results and assessing the practical value of the proposed module designs and extension of service lifetime. The described framework, along with the Tables S1–S3 (Supporting Information), can be applied for a replication, or can be adapted for other scenarios involving design alternatives and enhancements to the PV plant commissioning, operation, and electricity generation processes, possibly beginning the analysis from upstream components.

## Conflict of Interest

The authors declare no conflict of interest.

## Supporting information

Supporting Information

## Data Availability

The data that support the findings of this study are available from the corresponding author upon reasonable request.
