# Supplementary material for: Assessing the Environmental Benefits of Extending the Service Lifetime of Solar Photovoltaic Modules
Source: Glob Chall. 2024 Jul 16;8(8):2300245. doi: 10.1002/gch2.202300245 (PMC11316246; doi:10.1002/gch2.202300245)
Supplement: Supplementary file 1 — Supporting Information [file GCH2-8-2300245-s001.docx]

Supporting Information

Assessing the Environmental Benefits of Extending the Service Lifetime of Solar Photovoltaic Modules

Ahmed Burak PAC^*^, Abdulkerim GOK

The structure of this Supporting Information is organized as follows. Section S1 details the Life Cycle Inventories (LCI) of PV power plants with a service lifetime of 30 years. Section S2 expands on this foundation by presenting an extensive Life Cycle Assessment (LCA) of these plants, evaluating the environmental impacts of multi-crystalline and mono-crystalline modules, along with end-of-life treatment, and determining the contributions of each module and system components. In Section S3, PV modules that use the standard Ethylene-Vinyl Acetate (EVA) encapsulant are compared to those with alternative encapsulant materials under a 40-year optimistic scenario, evaluating potential benefits or burdens due to the use of different polymeric materials for module lamination. Section S4 describes the workflow employed for the statistical analysis of the Monte Carlo simulations. Lastly, Section S5 reports the results on the one-way ANOVA, subsequent Tukey Test, and Cohen’s D effect sizes.

**S1. Life Cycle Inventories (LCI)** **of PV Power Plants with a Service Lifetime of 30** **Years**

**Table S1.** Input and output flows for 1 m^2^ of mono-crystalline PV module.

| **Input** | **EVA** | **Alternative** | **Unit** |
| --- | --- | --- | --- |
| 1-propanol | 1.59E-02 | 1.59E-02 | kg |
| Aluminum alloy, AlMg_3_ | 2.13E+00 | 2.13E+00 | kg |
| Copper, cathode | 1.03E-01 | 1.03E-01 | kg |
| Corrugated cardboard box | 7.63E-01 | 7.63E-01 | kg |
| Diesel, burnt in building machine | 8.75E-03 | 8.75E-03 | MJ |
| Diode, auxiliaries, and energy use | 2.81E-03 | 2.81E-03 | kg |
| Electricity, medium voltage | 1.40E+01 | 1.40E+01 | kWh |
| Ethylene-vinyl acetate, foil | 8.75E-01 | 0.00E+00 | kg |
| EUR-flat pallet | 5.00E-02 | 5.00E-02 | units |
| Fiberglass reinforced plastic, polyamide,  injection molding | 2.95E-01 | 2.95E-01 | kg |
| Hydrogen fluoride | 6.24E-02 | 6.24E-02 | kg |
| Isopropanol | 1.47E-04 | 1.47E-04 | kg |
| Lead | 7.25E-04 | 7.25E-04 | kg |
| Lubricating oil | 1.61E-03 | 1.61E-03 | kg |
| PV cell, mono-Si wafer | 9.35E-01 | 9.35E-01 | m^2^ |
| PV module factory | 4.00E-06 | 4.00E-06 | units |
| High density polyethylene, granulate (PE-HD) | 2.38E-02 | 2.38E-02 | kg |
| Polyethylene terephthalate, granulated  (for the backsheet layer) | 3.46E-01 | 3.46E-01 | kg |
| Polyethylene terephthalate, granulated  (approximated for POE or TPO) | 0.00E+00 | 8.75E-01 | kg |
| Polyvinyl fluoride, film | 1.12E-01 | 1.12E-01 | kg |
| Potassium hydroxide | 5.14E-02 | 5.14E-02 | kg |
| Silicone product | 1.22E-01 | 1.22E-01 | kg |
| Soap | 1.16E-02 | 1.16E-02 | kg |
| Solar glass, low iron | 8.81E+00 | 8.81E+00 | kg |
| Tap water | 5.03E+00 | 5.03E+00 | kg |
| Tempering, flat glass | 8.81E+00 | 8.81E+00 | kg |
| Tin | 1.29E-02 | 1.29E-02 | kg |
| Transport, freight train | 1.66E+01 | 1.66E+01 | t×km |
| Transport, freight, lorry, 16-32 metric tons, EURO4 | 2.77E+00 | 2.77E+00 | t×km |
| Water | 5.03E-01 | 5.03E-01 | kg |
| Wire drawing, copper | 1.03E-01 | 1.03E-01 | kg |
|  | | | |
| **Output** | **EVA** | **Alternative** | **Unit** |
| PV module, mono-Si | 1.00E+00 | 1.00E+00 | m^2^ |
| Heat, waste | 5.03E+01 | 5.03E+01 | MJ |
| Carbon dioxide, fossil | 2.18E-02 | 2.18E-02 | kg |
| NMVOC, unknown origin | 8.06E-03 | 8.06E-03 | kg |
| Municipal solid waste | 3.00E-02 | 3.00E-02 | kg |
| Incineration, municipal solid waste* | -3.00E-02 | -3.00E-02 | kg |
| Waste plastics, mixture | 2.81E-02 | 2.81E-02 | kg |
| Incineration, plastics (PET, PMMA, PC)* | -2.81E-02 | -2.81E-02 | kg |
| Waste polyvinyl fluoride | 4.29E-03 | 4.29E-03 | kg |
| Incineration, plastic (hard PVC)* | -4.29E-03 | -4.29E-03 | kg |
| Hazardous waste, to incineration | 1.61E-03 | 1.61E-03 | kg |
| Residential wastewater | 4.53E-03 | 4.53E-03 | m^3^ |

^*Negative values represent waste management processes that accompany the waste output.^

**Table S2.** Input and output flows for commissioning open-ground 570 kWp mono-crystalline PV power plant.

| **Input** | **Amount** | **Unit** |
| --- | --- | --- |
| Inverter, 500 kW | 2.28E+00 | units |
| PV module, mono-Si | 2.94E+03 | m^2^ |
| PV mounting system, 570 kWp | 2.85E+03 | m^2^ |
| Electrical installation, 570 kWp | 9.30E-01 | units |
| Diesel, burnt in building machine | 6.96E+03 | MJ |
| Electric, low voltage | 3.27E+01 | kWh |
| Transport, lorry, 7.5-16 metric tons, fleet average | 8.34E+03 | t×km |
| Waste treatment, crystalline-Si PV module | 3.88E+04 | kg |
|  | | |
| **Output** | **Amount** | **Unit** |
| PV open-ground power plant, 570kWp | 1.00E+00 | units |
| Waste, heat | 1.18E+02 | MJ |

**Table S3.** Input and output flows for operating open-ground 570 kWp mono-crystalline PV power plant.

| **Input** | **Amount** | **Unit** |
| --- | --- | --- |
| PV open-ground power plant, 570kWp | 3.90E-8 | units |
| Tap water | 5.00E-3 | kg |
|  | | |
| **Output** | **Amount** | **Unit** |
| Electric, low voltage | 1.00E+00 | kWh |
| Waste, heat | 2.50E-01 | MJ |
| Waste, water | 5.00E-6 | m^3^ |

S2. Life Cycle Assessment (LCA) of PV Power Plants with a Service Lifetime of 30 Years

The environmental impacts of 1 kWh electricity generation from the 570 kWp mono-crystalline PV power plant, located in Central Anatolia (40^o^ North) with 1500 kWh/kWp/year performance output, are provided in **Table S4** in terms of ReCiPe midpoint categories. It is to be noted that 30 years of service life is considered here with no end-of-life treatment.

**Table S4.** Environmental impacts of 1 kWh electricity generation in terms of ReCiPe midpoint impact categories from the 570 kWp mono-crystalline PV power plant

| **Impact Category** | **Abbreviation** | **Impact Result** | **Unit** |
| --- | --- | --- | --- |
| Fine particulate matter formation | FMFP | 8.77E-05 | kg PM2.5-eq |
| Fossil resource scarcity | FFP | 1.05E-02 | kg oil-eq |
| Freshwater ecotoxicity | FETP | 8.41E-03 | kg 1,4DCB-eq |
| Freshwater eutrophication | FEP | 2.16E-05 | kg P-eq |
| Global warming potential | GWP | 3.75E-02 | kg CO_2_-eq |
| Human carcinogenic toxicity | HTPc | 6.29E-01 | kg 1,4DCB-eq |
| Human non-carcinogenic toxicity | HTPnc | 2.05E+01 | kg 1,4DCB-eq |
| Ionizing radiation | IRP | 4.84E-03 | kBq Co-60-eq |
| Land use | LOP | 1.25E-02 | m^2^·yr |
| Marine ecotoxicity | METP | 2.63E+01 | kg 1,4DCB-eq |
| Marine eutrophication | MEP | 3.07E-06 | kg N-eq |
| Mineral resource scarcity | SOP | 7.80E-04 | kg Cu-eq |
| Ozone formation, human health | HOFP | 1.10E-04 | kg NO_x_-eq |
| Ozone formation, terrestrial ecosystems | EOFP | 1.10E-04 | kg NO_x_-eq |
| Stratospheric ozone depletion | ODP | 2.45E-08 | kg CFC-11-eq |
| Terrestrial acidification | TAP | 1.60E-04 | kg SO_2_-eq |
| Terrestrial ecotoxicity | TETP | 6.83E-01 | kg 1,4DCB-eq |
| Water consumption | WCP | 1.00E-03 | m^3^ |

The results presented in Table S4 are compared with a 30-year 570 kWp multi-crystalline PV power plant in **Figure S1**. For this, the input already available in the ecoinvent database was used with slight modifications of the flows that were reported in Table S1, Table S2, and Table S3. As the unit process for the multi-crystalline technology available in the ecoinvent database did not consider end-of-life treatment, it was not incorporated into the model presented in Figure S1. However, waste prevention and recycling processes are available for the mono-crystalline technology in newer life cycle inventories, and therefore, it was included in Figure S1 for comparison. It is to be noted that recycling processes here only deal with glass, metal, and some polymeric materials, involved in both module and balance-of-system components, but not with solar cells, impact of which on the global warming potential comprise more than 60 % of the total burden, EVA encapsulants due to their crosslinked nature, and backsheets due to their multi-layer structure.


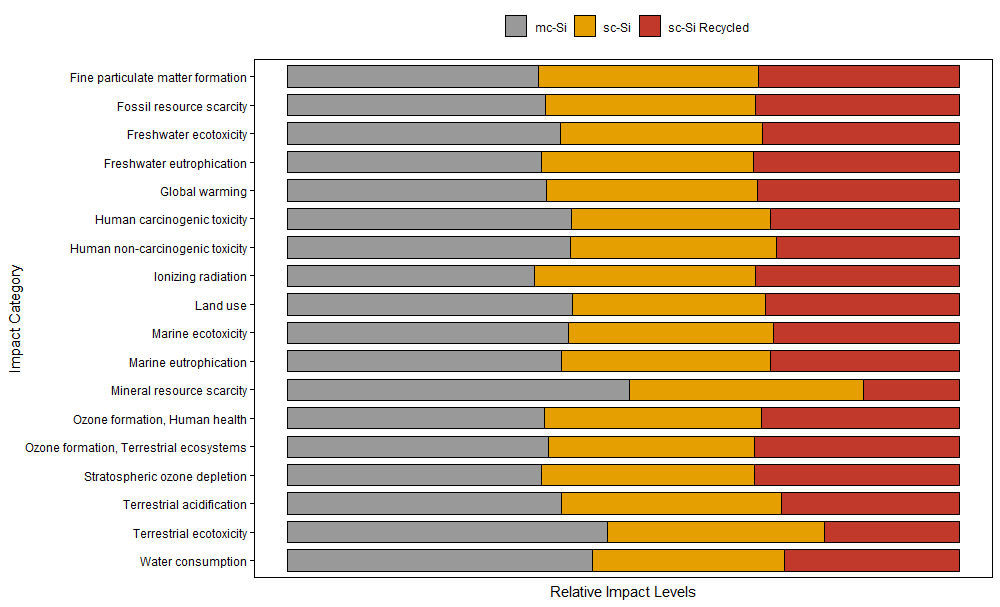


**Figure S1.** Environmental impacts of 1 kWh electricity generation in terms of ReCiPe midpoint impact categories from the 570 kWp PV power plants with mono-crystalline, recycled mono-crystalline, and multi-crystalline modules, with 1500 kWh/kWp/year performance output with a service lifetime of 30 years.

For the same installation capacity, mono-crystalline modules with higher module efficiencies led to less module area, mounting system needs, and cable usage compared to multi-crystalline modules, and thus, resulted in lower environmental impacts in almost all categories. When compared the mono-crystalline modules with and without recycling, recycling led to lower impacts in almost all categories. More evident impact reductions were observed in mineral resource scarcity (59%), terrestrial ecotoxicity (38%), terrestrial acidification (19%), and human non-carcinogenic toxicity (12%). A reduction of 3% to 9% was observed in global warming potential and many other categories.

The contributions of PV module and system components to global warming potential, mineral resource scarcity, and terrestrial acidification are shown in **Figure S2**. The production of solar cells, along with mounting systems, dominates all three impact categories. Metallic material consumption in mounting systems, module frames, cell metallization, inverters, and cables/wires for electrical installation account for most of the contributions to mineral resource scarcity.


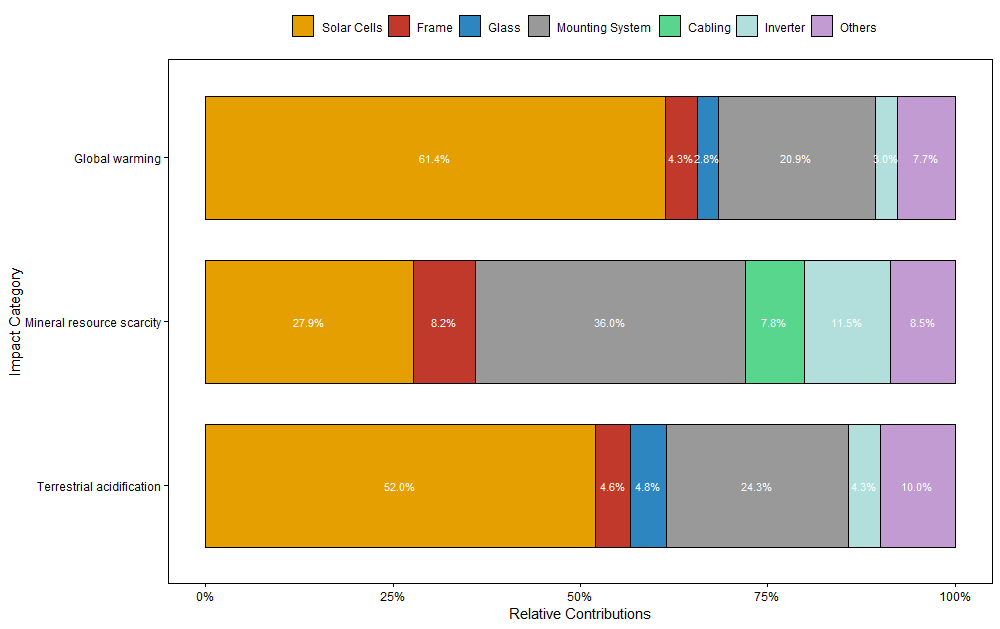


**Figure S2.** Relative contributions of module and system components to the impacts of global warming, mineral resource scarcity, and terrestrial acidification for 1 kWh electricity generation from the 570 kWp mono-crystalline power plant.

These findings highlight the influence of module and systems components on the environmental impacts. Therefore, ensuring the durability and reliability of modules for long-term operation is of vital importance in reducing life cycle impacts. The selection of alternative materials for an improved lamination process, with increased recyclability, can contribute to more sustainable modules. This, in turn, enables power plants to operate at high performance levels for extended periods, leading to higher energy output from the limited resources used during the production of modules and commissioning of the power plant.

**S3. Comparison of** **modules with current standard EVA and alternative encapsulant materials under the 40-Year** **Optimistic Scenario**

**Table S5.** Environmental impacts of 1 kWh electricity generation in terms of ReCiPe midpoint impact categories from the 570 kWp PV power plants with modules laminated with current standard EVA and alternative encapsulant materials with a service lifetime of 40 years under the optimistic scenario.

| **Impact Category*** | **40-Year EVA** | **40-Year Alternative** | **Unit** |
| --- | --- | --- | --- |
| Fine particulate matter formation | 6.05E-05 | 6.06E-05 | kg PM2.5-eq |
| Fossil resource scarcity | 7.67E-03 | 7.66E-03 | kg oil-eq |
| Freshwater ecotoxicity | 6.96E-03 | 6.97E-03 | kg 1,4DCB-eq |
| Freshwater eutrophication | 1.62E-05 | 1.62E-05 | kg P-eq |
| Global warming potential | 2.72E-02 | 2.72E-02 | kg CO_2_-eq |
| Human carcinogenic toxicity | 4.53E-01 | 4.54E-01 | kg 1,4DCB-eq |
| Human non-carcinogenic toxicity | 1.45E+01 | 1.45E+01 | kg 1,4DCB-eq |
| Ionizing radiation | 3.38E-03 | 3.37E-03 | kBq Co-60-eq |
| Land use | 9.39E-03 | 9.39E-03 | m^2^·yr |
| Marine ecotoxicity | 1.92E+01 | 1.92E+01 | kg 1,4DCB-eq |
| Marine eutrophication | 2.10E-06 | 2.10E-06 | kg N-eq |
| Mineral resource scarcity | 2.60E-04 | 2.60E-04 | kg Cu-eq |
| Ozone formation, human health | 7.77E-05 | 7.75E-05 | kg NO_x_-eq |
| Ozone formation, terrestrial ecosystems | 8.08E-05 | 8.06E-05 | kg NO_x_-eq |
| Stratospheric ozone depletion | 1.78E-08 | 1.92E-08 | kg CFC-11-eq |
| Terrestrial acidification | 9.86E-05 | 9.87E-05 | kg SO_2_-eq |
| Terrestrial ecotoxicity | 3.23E-01 | 3.23E-01 | kg 1,4DCB-eq |
| Water consumption | 6.80E-04 | 6.80E-04 | m^3^ |

^Except for the stratospheric ozone depletion, all impact categories between the two module construction experience differences of less than 1%. Regarding the stratospheric ozone depletion, the 7.4% difference could be attributed to the very low absolute magnitude of the values involved.^


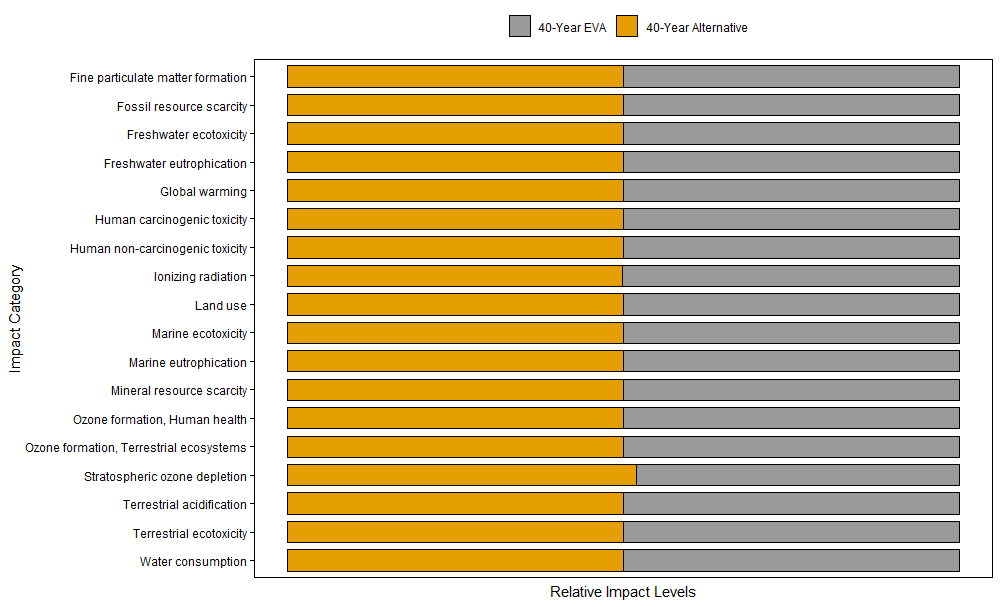


**Figure S3.** Comparison of environmental impacts of 1 kWh electricity generation in terms of ReCiPe midpoint impact categories from the 570 kWp PV power plants with modules laminated with current standard EVA and alternative encapsulant materials with a service lifetime of 40 years under the optimistic scenario.

**S4. Analysis Workflow for the Monte Carlo simulations**


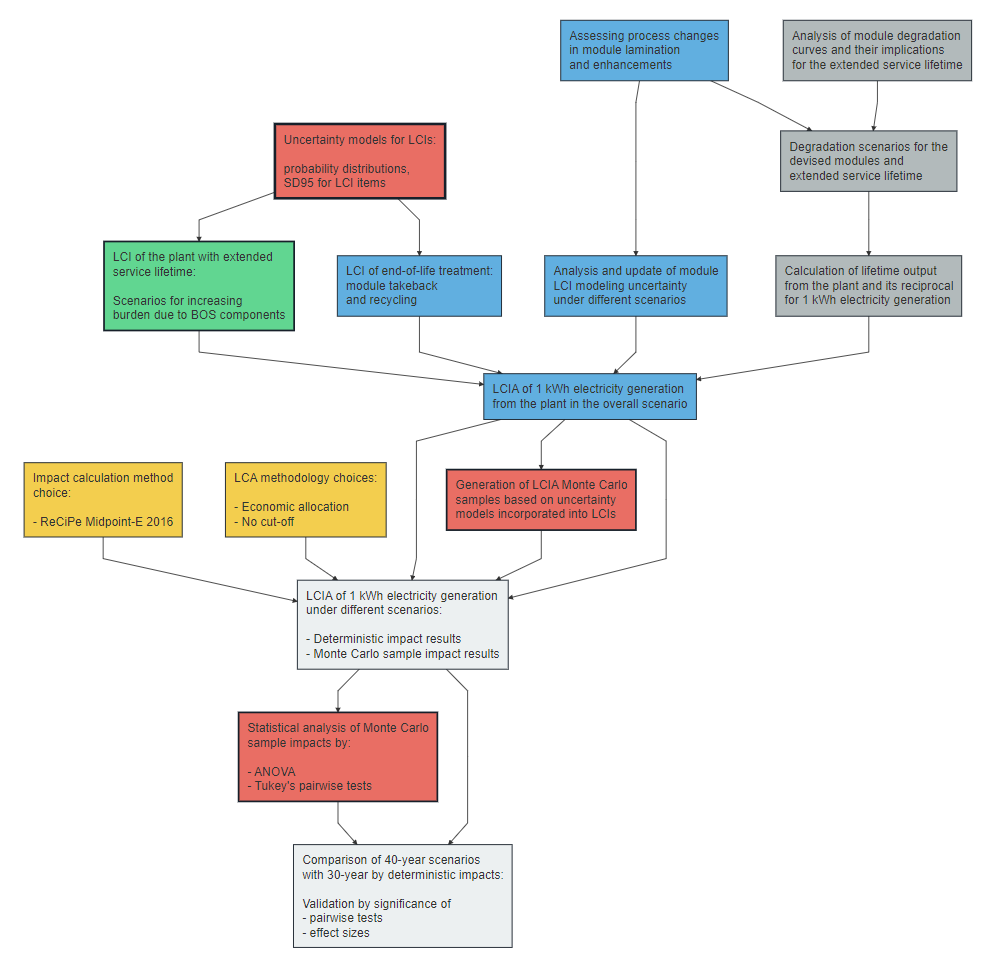


**Figure S4.** Workflow of the analytical framework applied in this study. This framework examines alternative lamination processes, degradation, and the growing burden on PV plant components during extended service lifetime. It involves data collection and LCI compilation, considering various scenarios that reflect the uncertainties associated with alternative processes and prolonged service lifetimes. LCA is then set up to calculate impacts and facilitate detailed result analysis.

**S5. Mean/Standard-Deviations, One-way ANOVA, Subsequent Tukey Test, and Cohen’s D Effect Sizes of the Monte Carlo Samples Obtained for the ReCiPe Midpoint Impact Categories for 30-Year and 40-Year Scenarios**

**Table S6.** Mean and standard deviations for ReCiPe Midpoint environmental impact categories on Monte Carlo samples (n=60) generated for the 570 kWp PV power plants with a service lifetime of 30 years and 40 years under different scenarios.

| **Impact category** | **30-Year** | | **40-Year**  **Optimistic** | | **40-Year**  **Moderate** | | **40-Year**  **Pessimistic** | | **40-Year**  **Pessimistic Efficient** | |
| --- | --- | --- | --- | --- | --- | --- | --- | --- | --- | --- |
|  | **Mean** | **SD** | **Mean** | **SD** | **Mean** | **SD** | **Mean** | **SD** | **Mean** | **SD** |
| FMFP | 8.15E-05 | 5.64E-06 | 6.12E-05 | 4.20E-06 | 6.28E-05 | 4.43E-06 | 6.37E-05 | 4.50E-06 | 6.12E-05 | 4.36E-06 |
| FFP | 1.03E-02 | 7.31E-04 | 7.74E-03 | 5.45E-04 | 7.93E-03 | 5.75E-04 | 8.05E-03 | 5.85E-04 | 7.73E-03 | 5.63E-04 |
| FETP | 9.41E-03 | 3.14E-04 | 7.06E-03 | 2.35E-04 | 7.24E-03 | 2.47E-04 | 7.34E-03 | 2.48E-04 | 7.06E-03 | 2.42E-04 |
| FEP | 2.18E-05 | 1.37E-06 | 1.64E-05 | 1.02E-06 | 1.68E-05 | 1.07E-06 | 1.70E-05 | 1.10E-06 | 1.63E-05 | 1.06E-06 |
| GWP | 3.65E-02 | 2.53E-03 | 2.74E-02 | 1.89E-03 | 2.81E-02 | 1.99E-03 | 2.85E-02 | 2.03E-03 | 2.74E-02 | 1.95E-03 |
| HTPc | 6.07E-01 | 2.07E-02 | 4.56E-01 | 1.57E-02 | 4.67E-01 | 1.63E-02 | 4.74E-01 | 1.65E-02 | 4.55E-01 | 1.61E-02 |
| HTPnc | 1.96E+01 | 1.05E+00 | 1.47E+01 | 7.92E-01 | 1.51E+01 | 8.25E-01 | 1.53E+01 | 8.39E-01 | 1.47E+01 | 8.19E-01 |
| IRP | 4.54E-03 | 3.85E-04 | 3.41E-03 | 2.87E-04 | 3.50E-03 | 3.02E-04 | 3.55E-03 | 3.08E-04 | 3.41E-03 | 2.97E-04 |
| LOP | 1.25E-02 | 7.12E-05 | 9.40E-03 | 5.33E-05 | 9.64E-03 | 5.58E-05 | 9.77E-03 | 5.57E-05 | 9.40E-03 | 5.49E-05 |
| METP | 2.59E+01 | 1.28E+00 | 1.94E+01 | 9.60E-01 | 1.99E+01 | 1.00E+00 | 2.02E+01 | 1.02E+00 | 1.94E+01 | 9.94E-01 |
| MEP | 2.82E-06 | 2.55E-07 | 2.12E-06 | 1.90E-07 | 2.17E-06 | 2.00E-07 | 2.20E-06 | 2.04E-07 | 2.12E-06 | 1.97E-07 |
| SOP | 3.49E-04 | 3.61E-05 | 2.64E-04 | 2.70E-05 | 2.70E-04 | 2.79E-05 | 2.74E-04 | 2.81E-05 | 2.63E-04 | 2.83E-05 |
| HOFP | 1.04E-04 | 6.65E-06 | 7.80E-05 | 4.98E-06 | 8.00E-05 | 5.24E-06 | 8.12E-05 | 5.33E-06 | 7.80E-05 | 5.14E-06 |
| EOFP | 1.08E-04 | 6.98E-06 | 8.11E-05 | 5.22E-06 | 8.32E-05 | 5.49E-06 | 8.44E-05 | 5.59E-06 | 8.10E-05 | 5.39E-06 |
| ODP | 2.40E-08 | 1.78E-09 | 1.80E-08 | 1.32E-09 | 1.85E-08 | 1.40E-09 | 1.87E-08 | 1.42E-09 | 1.80E-08 | 1.37E-09 |
| TAP | 1.32E-04 | 9.26E-06 | 9.95E-05 | 6.93E-06 | 1.02E-04 | 7.27E-06 | 1.04E-04 | 7.37E-06 | 9.94E-05 | 7.17E-06 |
| TETP | 4.34E-01 | 6.63E-02 | 3.27E-01 | 4.94E-02 | 3.35E-01 | 5.18E-02 | 3.40E-01 | 5.29E-02 | 3.26E-01 | 5.14E-02 |
| WCP | 9.18E-04 | 8.84E-05 | 6.90E-04 | 6.58E-05 | 7.07E-04 | 6.94E-05 | 7.17E-04 | 7.08E-05 | 6.89E-04 | 6.82E-05 |

**Table S7.** The results of one-way ANOVA test with p-values for the ReCiPe Midpoint environmental impact categories on Monte Carlo samples (n=60) generated for the 570 kWp PV power plants with a service lifetime of 30 years and 40 years under different scenarios, followed by subsequent Tukey pairwise scenario comparisons for the 40-year scenarios.

| **Impact Category** | **ANOVA** | **40-Year Optimistic vs.**  **40-Year**  **Moderate** | **40-Year Optimistic vs.**  **40-Year Pessimistic** | **40-Year Optimistic vs.**  **40-Year Pessimistic Efficient** | **40-Year Moderate vs.**  **40-Year Pessimistic** | **40-Year Moderate vs.**  **40-Year Pessimistic Efficient** | **40-Year Pessimistic vs.**  **40-Year Pessimistic Efficient** |
| --- | --- | --- | --- | --- | --- | --- | --- |
| FMFP | *4.81E-87* | 5.43E-01 | *8.42E-06* | 1.00E+00 | *6.20E-03* | 5.46E-01 | *8.60E-06* |
| FFP | *1.61E-84* | 5.57E-01 | *1.05E-05* | 1.00E+00 | *6.71E-03* | 5.43E-01 | *9.41E-06* |
| FETP | *2.38E-168* | *2.15E-06* | *3.49E-13* | 7.35E-01 | 1.28E-01 | *7.22E-04* | *2.68E-09* |
| FEP | *1.66E-96* | 3.34E-01 | *1.59E-06* | 1.00E+00 | *6.72E-03* | 3.69E-01 | *2.22E-06* |
| GWP | *2.99E-86* | 5.42E-01 | *8.59E-06* | 1.00E+00 | *6.32E-03* | 5.34E-01 | *8.04E-06* |
| HTPc | *8.42E-163* | *5.12E-03* | *1.98E-12* | 1.00E+00 | *1.02E-03* | *4.08E-03* | *1.21E-12* |
| HTPnc | *3.11E-113* | 9.07E-02 | *1.24E-06* | 1.00E+00 | *4.25E-02* | 8.90E-02 | *1.19E-06* |
| IRP | *2.79E-68* | 7.25E-01 | *8.99E-05* | 1.00E+00 | *1.24E-02* | 6.90E-01 | *6.91E-05* |
| LOP | 0.00E+00 | 0.00E+00 | 0.00E+00 | 9.99E-01 | 0.00E+00 | 0.00E+00 | 0.00E+00 |
| METP | *1.19E-122* | *3.96E-02* | *1.32E-07* | 1.00E+00 | *3.33E-02* | 5.46E-02 | *2.58E-07* |
| MEP | *2.04E-63* | 8.08E-01 | *2.49E-04* | 1.00E+00 | *1.60E-02* | 7.38E-01 | *1.43E-04* |
| SOP | *1.74E-53* | 9.10E-01 | 3.25E-01 | 9.24E-01 | 8.45E-01 | 4.44E-01 | 5.32E-02 |
| HOFP | *4.34E-94* | 4.21E-01 | *2.75E-06* | 1.00E+00 | *5.72E-03* | 4.30E-01 | *2.97E-06* |
| EOFP | *3.03E-93* | 4.31E-01 | *3.04E-06* | 1.00E+00 | *5.75E-03* | 4.39E-01 | *3.24E-06* |
| ODP | *3.13E-80* | 6.13E-01 | *2.55E-05* | 1.00E+00 | *9.23E-03* | 5.90E-01 | *2.15E-05* |
| TAP | *5.44E-86* | 5.49E-01 | *3.51E-05* | 1.00E+00 | *1.59E-02* | 4.59E-01 | *1.79E-05* |
| TETP | *5.60E-30* | 9.90E-01 | *2.22E-02* | 9.93E-01 | 8.36E-02 | 8.94E-01 | *5.28E-03* |
| WCP | *3.99E-58* | 8.51E-01 | *3.83E-04* | 1.00E+00 | *1.67E-02* | 7.76E-01 | *2.02E-04* |

^*Significant ANOVA and Tukey test results, with a p value of ≤0.05, are shown in italic face, while non-significant results are shown in normal face. The Tukey test results for all comparisons for the 30-year scenario with other scenarios are 0, thus not included.^

**Table S8.** Cohen’s D effect sizes of the Monte Carlo samples (n=60) obtained for the ReCiPe Midpoint impact categories for pairwise comparisons for the 40-year scenarios with the 30-year scenario.

| **Impact Categories*** | **30-Year**  **vs.**  **40-Year Optimistic** | **30-Year**  **vs.**  **40-Year Moderate** | **30-Year**  **vs.**  **40-Year Pessimistic** | **30-Year**  **vs.**  **40-Year Pessimistic Efficient** |
| --- | --- | --- | --- | --- |
| FMFP | 3.97 | 3.79 | 3.18 | 4.20 |
| FFP | 3.84 | 3.67 | 3.06 | 4.07 |
| FETP | 8.56 | 7.85 | 7.57 | 8.83 |
| FEP | 4.31 | 4.13 | 3.54 | 4.59 |
| GWP | 3.91 | 3.74 | 3.12 | 4.14 |
| HTPc | 7.91 | 7.53 | 6.94 | 8.39 |
| HTPnc | 5.07 | 4.85 | 4.42 | 5.46 |
| IRP | 3.18 | 3.06 | 2.49 | 3.41 |
| LOP | 50.69 | 49.33 | 47.10 | 52.50 |
| METP | 5.54 | 5.28 | 4.85 | 5.94 |
| MEP | 3.01 | 2.90 | 2.35 | 3.23 |
| SOP | 2.70 | 2.50 | 2.57 | 2.95 |
| HOFP | 4.29 | 4.07 | 3.42 | 4.51 |
| EOFP | 4.25 | 4.03 | 3.39 | 4.48 |
| ODP | 3.65 | 3.50 | 2.92 | 3.87 |
| TAP | 3.95 | 3.77 | 3.19 | 4.20 |
| TETP | 1.76 | 1.74 | 1.31 | 1.97 |
| WCP | 2.79 | 2.70 | 2.16 | 3.01 |

**Table S9.** Cohen’s D effect sizes of the Monte Carlo samples (n=60) obtained for the ReCiPe Midpoint impact categories for pairwise comparisons for the 40-year scenarios.

| **Impact Categories** | **40-Year Optimistic vs.**  **40-Year**  **Moderate** | **40-Year Optimistic vs.**  **40-Year Pessimistic** | **40-Year Optimistic vs.**  **40-Year Pessimistic Efficient** | **40-Year Moderate vs.**  **40-Year Pessimistic** | **40-Year Moderate vs.**  **40-Year Pessimistic Efficient** | **40-Year Pessimistic vs.**  **40-Year Pessimistic Efficient** |
| --- | --- | --- | --- | --- | --- | --- |
| FMFP | 0.30 | 0.93 | 0.00 | 0.66 | 0.32 | 1.01 |
| FFP | 0.29 | 0.92 | 0.00 | 0.66 | 0.32 | 1.01 |
| FETP | 0.97 | 1.43 | 0.24 | 0.46 | 0.79 | 1.29 |
| FEP | 0.36 | 0.99 | 0.01 | 0.67 | 0.38 | 1.08 |
| GWP | 0.30 | 0.93 | 0.00 | 0.66 | 0.33 | 1.01 |
| HTPc | 0.65 | 1.40 | 0.01 | 0.76 | 0.72 | 1.53 |
| HTPnc | 0.47 | 1.00 | 0.00 | 0.55 | 0.52 | 1.11 |
| IRP | 0.24 | 0.84 | 0.01 | 0.62 | 0.28 | 0.93 |
| LOP | 3.86 | 6.44 | 0.04 | 2.70 | 4.06 | 6.74 |
| METP | 0.53 | 1.07 | 0.02 | 0.57 | 0.56 | 1.17 |
| MEP | 0.21 | 0.79 | 0.03 | 0.61 | 0.26 | 0.90 |
| SOP | 0.15 | 0.37 | 0.16 | 0.20 | 0.31 | 0.57 |
| HOFP | 0.34 | 0.97 | 0.00 | 0.66 | 0.36 | 1.04 |
| EOFP | 0.33 | 0.97 | 0.00 | 0.66 | 0.36 | 1.04 |
| ODP | 0.28 | 0.89 | 0.01 | 0.64 | 0.31 | 0.98 |
| TAP | 0.29 | 0.87 | 0.03 | 0.60 | 0.35 | 0.96 |
| TETP | 0.09 | 0.57 | 0.09 | 0.50 | 0.19 | 0.72 |
| WCP | 0.20 | 0.78 | 0.03 | 0.61 | 0.25 | 0.89 |


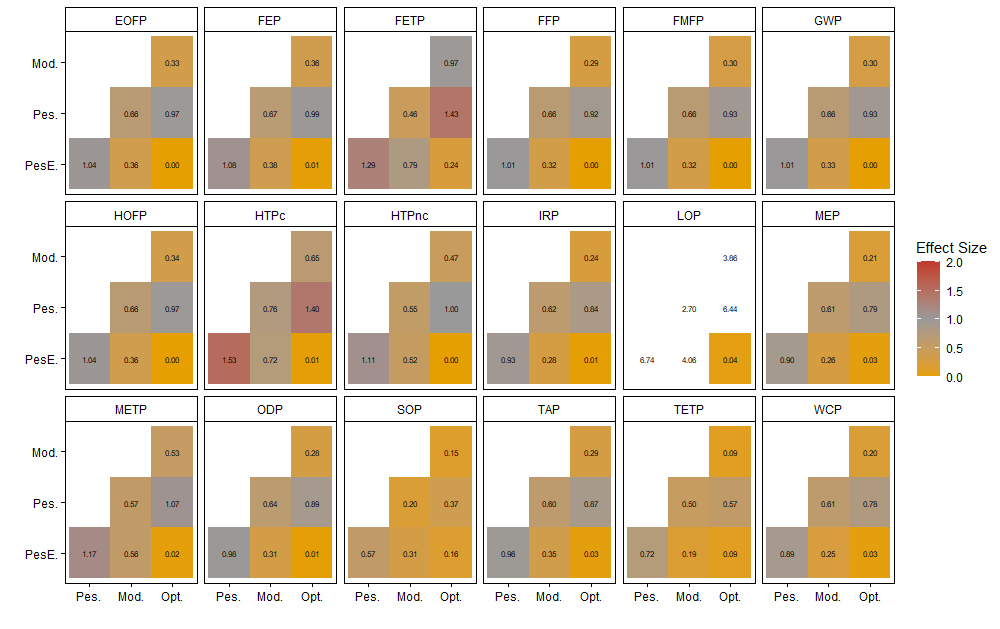


**Figure S5.** Heatmap of Cohen’s D effect sizes of the Monte Carlo samples (n=60) obtained for the ReCiPe Midpoint impact categories for pairwise comparisons for the 40-year scenarios.
